# Underestimated effect of intragenic HIV-1 DNA methylation on viral transcription in infected individuals

**DOI:** 10.1186/s13148-020-00829-1

**Published:** 2020-02-28

**Authors:** Sam Kint, Wim Trypsteen, Ward De Spiegelaere, Eva Malatinkova, Sabine Kinloch-de Loes, Tim De Meyer, Wim Van Criekinge, Linos Vandekerckhove

**Affiliations:** 1 HIV Cure Research Center, Department of Internal Medicine and Pediatrics, Faculty of Medicine and Health Sciences, Ghent University and Ghent University Hospital, Corneel Heymanslaan 10, Medical Research Building 2, 9000 Ghent, Belgium; 2 Biobix, Department of Data Analysis and Mathematical Modelling, Faculty of Bio-science Engineering, Ghent University, Coupure Links 653, 9000 Ghent, Belgium; 3 Department of Morphology, Faculty of Veterinary Medicine, Ghent University, Salisburylaan 133, 9820 Merelbeke, Belgium; 4 Division of Infection and Immunity, Royal Free Hospital, Royal Free Campus, University College London, Pont St, Hampstead, London, NW3 2QG UK

**Keywords:** HIV-1, DNA methylation, HIV-1 latency, Epigenetics, Next-generation sequencing, Bisulfite sequencing, Intragenic DNA methylation

## Abstract

**Background:**

The HIV-1 proviral genome harbors multiple CpG islands (CpGIs), both in the promoter and intragenic regions. DNA methylation in the promoter region has been shown to be heavily involved in HIV-1 latency regulation in cultured cells. However, its exact role in proviral transcriptional regulation in infected individuals is poorly understood or characterized. Moreover, methylation at intragenic CpGIs has never been studied in depth.

**Results:**

A large, well-characterized HIV-1 patient cohort (*n* = 72), consisting of 17 long-term non-progressors and 8 recent seroconverters (SRCV) without combination antiretroviral therapy (cART), 15 early cART-treated, and 32 late cART-treated patients, was analyzed using a next-generation bisulfite sequencing DNA methylation method. In general, we observed low level of promoter methylation and higher levels of intragenic methylation. Additionally, SRCV showed increased promoter methylation and decreased intragenic methylation compared with the other patient groups. This data indicates that increased intragenic methylation could be involved in proviral transcriptional regulation.

**Conclusions:**

Contrasting in vitro studies, our results indicate that intragenic hypermethylation of HIV-1 proviral DNA is an underestimated factor in viral control in HIV-1-infected individuals, showing the importance of analyzing the complete proviral genome in future DNA methylation studies.

## Background

Current combination antiretroviral therapy (cART) can successfully control human immunodeficiency virus type 1 (HIV-1) infection and prevent disease progression to the acquired immunodeficiency syndrome (AIDS). However, a cure is not generally achievable due to the establishment of a latent reservoir of proviral HIV-1 DNA which remains dormant and fuels viral rebound upon treatment interruption [1–4]. Therefore, better insight into the mechanisms regulating HIV-1 latency is crucial in order to interfere with this latency state and to develop cure strategies. The state of HIV-1 latency can be defined as the transcriptional silencing of proviral genes caused by multiple transcriptional blocks after the stable integration of proviral DNA into the host genome [5]. Some of the major silencing mechanisms consist of epigenetic modifications, which have led to several clinical trials investigating the latent viral reservoir reactivation with histone deacetylase inhibitors, albeit with limited success [6–10]. Other epigenetic modifications such as HIV-1 proviral DNA methylation have also been described in HIV-1 transcriptional silencing and have been explored as targets for HIV-1 latency reversing strategy [11–14].

DNA methylation is a well-described epigenetic modification in which a methyl group is added at the number five carbon of the cytosine pyrimidine ring in CpG dinucleotides [15, 16]. This modification plays a role in genome transcription regulation and is crucial in processes such as the development of multicellular organisms, cell differentiation, regulation of gene expression, X-chromosome inactivation, genomic imprinting, and in the suppression of parasitic and other repeat sequences [15–23]. In general, reliable and stable transcriptional silencing is caused if CpG islands (CpGIs)—stretches of DNA that contain an increased frequency of CpG dinucleotides (CG content > 50% and observed/expected CpG ratio > 60%)—in promoter regions are hypermethylated [12, 15, 16, 24, 25]. Methylation of CpGIs within gene bodies (intragenic methylation) has been shown to be involved in regulation of intragenic promoters, alternative splicing, and cellular differentiation, but also in the activation of retroviruses, repetitive elements, and prevention of aberrant transcript production [26–30].

**Fig. 1:**
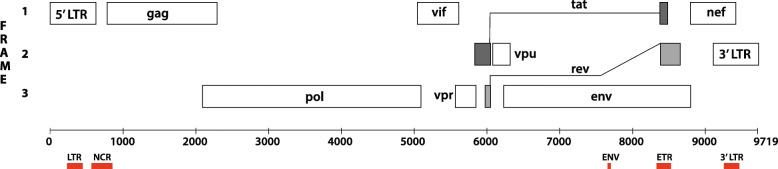
Location of the 5 CpGIs in the HIV-1 genome. The locations of the 5 CpGIs as described by Chavéz et al. [12] are indicated by red bars. CpGI long terminal repeat (LTR) and non-coding region (NCR) are located around the HIV-1 promoter location. CpGI ENV and *env-tat-rev* are located in the *env* gene. The fifth CpGI (3′ LTR) is located in the 3′ LTR region, where the antisense promoter region is found

The HIV-1 genome encodes five CpGIs [12]: two are surrounding the promoter region and flanking the HIV-1 transcription start site and several transcription factor binding sites (e.g., TCF-1α, NF-κB, SP1) at the 5′ long terminal repeat (LTR) region (CpGI LTR in the U3 region of the 5′ LTR and CpGI non-coding region (NCR), downstream the HIV-1 5′ LTR (Fig. 1)) [12]. Two other CpGIs are located in the *env* gene (CpGI ENV (35% conserved) and CpGI *env*-*tat*-*rev* (ETR)), surrounding the HIV-1 antisense open reading frame (Fig. 1) [12, 31]. The fifth CpGI is located in the 3′ LTR, where the antisense transcription start site is located [12, 31]. In cultured HIV-1-infected cells, the regulatory role of proviral promoter methylation in viral transcriptional activity is clearly demonstrated: hypermethylation stabilizes HIV-1 latency and demethylating agents can induce activation of HIV-1 transcription [12, 13, 32–34]. However, studies performed on DNA methylation in infected individuals could not reproduce these findings indicating that this in vitro regulation does not apply in vivo [14, 32, 35–38].

To further understand the role of proviral HIV-1 DNA methylation in infected individuals, an NGS-based bisulfite assay was developed to characterize HIV-1 proviral DNA methylation profiles of both promotor and intragenic regions in the context of a large, well-characterized patient cohort (*n* = 72). This cohort comprises four different patient groups as described by Malatinkova et al. [39]: 15 early cART-treated individuals (ET), 32 late cART-treated individuals (LT), 17 long-term non-progressors (LTNP), and 8 acute seroconverters (SRCV).

## Methods

### Patient cohorts and DNA samples

HIV-1-positive patients were recruited from two clinical centers, the Ian Charleson Day Centre (Royal Free Hospital, London, UK) and the AIDS Reference Center (Ghent University Hospital, Ghent, Belgium) during the study performed by Malatinkova et al. [39]. Seventy-two HIV-1-positive PBMC samples from that study were selected. Patients were divided into four cohorts based on their disease status (Additional Figure 1). The detailed study design and inclusion criteria have been described previously [39]. Briefly, (1) long-term cART-treated individuals (median treatment time of 10.77 years (interquartile range (IQR), 6.46–12.34 years)) who had initiated treatment during HIV-1 seroconversion (early treated (ET); *n* = 15) or (2) during the chronic phase of the infection (late treated (LT); *n* = 32); (3) cART-naïve long-term non-progressors (LTNPs, *n* = 17) who had maintained HIV-1 viral load (VL) ≤ 1000 copies/ml and CD4+ T cells > 500 cells/mm^3^ over > 7 years post-infection or (4) cART-naïve seroconverters (SRCV, *n* = 8), who were sampled during the acute phase of the infection. Baseline characteristics and clinical parameters of these cohorts are summarized in Table 1. The Ethical Committees of Ghent University Hospital and the Royal Free Hospital had approved this study (reference numbers: B670201317826 (Ghent) and 13/LO/0729 (London)) with all study subjects giving their written informed consent.

**Table 1:** Clinical characteristics and viral reservoir markers of the four patient cohorts

	Cohort 1 = ET	Cohort 2 = LTNP	Cohort 3 = LT	Cohort 4 = SRCV
# patients	15	17	32	8
Clinical characteristics				
Age (years)	45 (43–54.5)	49 (38–51)	48 (45–53.25)	37 (27–44.75)
Total cART (years)	11.65 (10.39–11.97)	0 (0–0)	9.80 (6.09–14.73)	0 (0–0)
Total VL suppression (years)	11.18 (9.82–11.37)	9.72 (0–14.67)	6.53 (5–10.42)	0 (0–0)
log VL zenith (copies/ml)	5.74 (5.31–5.88)	2.24 (1.79–2.76)	4.93 (4.24–5.52)	6.15 (5.14–6.31)
CD4 nadir (cells/μl)	413.5 (274.5–539.75)	624 (562–693)	154.5 (51.25–266.25)	483.5 (393.75–520.25)
CD4 at collection (cells/μl)	961 (737–1129.5)	793 (685–1010)	624.5 (484–885.5)	534 (393.75–617.50)
CD4/CD8	1.12 (0.8–1.47)	0.91 (0.82–1.47)	0.74 (0.6–0.93)	0.62 (0.37–0.87)
Viral reservoir markers				
Total HIV-1 DNA^*^ (c/M PBMC) [40]	88.14 (46.19–124.02)	48.01 (20.16–56.50)	137.01 (56.08–219.20)	1290.48 (519.63–4428.60)
Integrated HIV-1 DNA^*^ (c/M PBMC) [41, 42]	158.00 (122.70–388.55)	28.16 (0–158.41)	586.65 (315.12–918.15)	1802.68 (272.19–3966.55)
CA HIV-1 usRNA (c/M PBMC) [43]	0.79 (0.28–3.12)	0.44 (0.27–3.51)	6.12 (1.80–10.08)	15.47 (0.62–77.60)
2-LTR circles (c/M PBMC) [40]	1.48 (0–3.03)	0.77 (0.65–2.70)	1.32 (0.57–2.18)	15.35 (4.82–24.12)

Values are reported as median (interquartile range), *SRCV* seroconverters, *LTNP* long-term non-progressors, *PBMCs* peripheral blood mononuclear cells, *CA* cell-associated, *usRNA* unspliced RNA, *cART* combination antiretroviral therapy, *VL* viral load

^*^Total and integrated HIV-1 DNA measurements are performed using different assays and the absolute copies are therefore not directly comparable. To measure integrated HIV-1 DNA, an Alu-HIV-1 qPCR is used whereas digital PCR is used to determine the total number of HIV-1 DNA copies

DNA from aliquots of 10^7^ PBMCs was isolated using the DNeasy® Blood & Tissue Kit (Qiagen, The Netherlands, 69504). Sample DNA concentration was determined with the Qubit dsDNA BR (broad range) Assay Kit (Thermo Fisher Scientific, MA, USA, Q32850) on a Qubit 2.0 fluorometer according to the manufacturer’s instructions.

### Cell culture

Jurkat cells (human T cell leukemia line) and J-Lat 8.4 (Jurkat cells infected with one HIV-1 copy per cell [44]) were cultured in a humidified atmosphere of 37 °C and 5% CO_2_ in RPMI 1640 medium with GlutaMAX™ Supplement (Thermo Fisher Scientific, MA, USA, 61870-010), supplemented with 10% FCS and 100 μg/ml penicillin/streptomycin. The culture medium was renewed every 2 to 3 days. DNA was isolated as described in the previous section.

### Primer design

Primers targeting the 4 major HIV-1 CpGIs were designed using 2 online available primer design tools (Methprimer [45] and bisulfite primer seeker (Zymo Research, CA, USA, https://www.zymoresearch.com/pages/bisulfite-primer-seeker)). LTR primers were obtained from Trejbalova et al*.* [13] and ETR_1 primers from Weber et al*.* [37]. To evaluate primers in silico, the bio-informatics tool developed by Rutsaert et al*.* [46], estimating the complementarity of each primer combination to all full-length HIV-1 sequences in the Los Alamos National Laboratory (LANL) database (www.hiv.lanl.gov) [47], was adapted: the database was transformed to the bisulfite-treated variant (C→T; CG→CG), nested primer combination analysis was included, as well as analysis of combinations of multiple PCR assays. First, the in silico analysis was used to evaluate primer combinations that were obtained from literature as well as in-house designed. Primer combinations matching at least 50% of the LANL database and nested combinations with an overlap of at least 2/3 of the matched sequences were retained. Selected primers were in vitro tested using DNA from J-Lat 8.4 [44], diluted in Jurkat DNA at different concentrations to mimic patient samples (10,000, 5000, 1000, 500, 250, 100 HIV-1 copies per 10^6^ cells). Finally, an additional in silico analysis was used to select 4 or less primer combinations per CpGI that targeted at least 60% of the LANL database. These final primer sequences are listed in Additional File 1.

### Bisulfite treatment

A minimum of 5 × 1 μg of DNA per patient was bisulfite treated using the Epitect Bisulfite kit (Qiagen, The Netherlands, 59110), which is the least fragmenting commercial bisulfite kit available, according to a previous in-house comparison [18]. We used the standard protocol as provided by the manufacturer. The five aliquots per patient were pooled, and immediately stored at – 20 °C.

### Bisulfite-specific PCR

All PCR reactions were performed in triplicate to reduce the probability of preferential amplification of one specific amplicon that would dominate the output. Nested PCR reactions were performed using the FastStart™ Taq DNA Polymerase, 5 U/μl (Roche Applied Science, Belgium, 12032953001). A volume containing theoretically at least ten bisulfite-treated HIV-1 copies (based on the droplet digital PCR measurements as in Malatinkova et al. [39]) was added to the PCR mix containing 10 × PCR buffer, 2.5 U polymerase, 400 nM forward and reverse primers, and 3% DMSO in a final volume of 25 μl. Each CpGI was amplified with one nested primer combination, and after a failed PCR reaction, the subsequent primer combination was used (Additional File 1). Amplicons were visualized using 3% agarose gel electrophoresis. Depending on the selected primer, we used an in-house optimized PCR amplification protocol or one of the two previously published protocols [13, 37], as described in Additional File 1.

### Sequencing

Bisulfite-treated amplicons were pooled equimolarly and libraries were prepared using the NEBNext UltraII DNA Library Prep Kit for Illumina (NEB, MA, USA, #E7645L/#E7103L). These libraries were sequenced on a MiSeq sequencing system (MiSeq® Reagent Kit v3 (600 cycle), MS-102-3003, Illumina). Sequencing reads were trimmed using Trimmomatic (version 0.38), quality controlled using FastQC (version 0.11.8), and subsequently mapped to an in-house developed HIV-1 consensus genome using the Bismark package (version 0.10.1) [48], providing a conversion efficiency estimation and methylation state of all analyzed CpGs.

### Statistical analysis

HIV-1-specific amplicons with coverage > 250 were normalized and divided into tiles (blocks of the HIV-1 genome containing the region of interest (LTR or *env*)). Differential methylation analysis per region was performed using the MethylKit package (version 1.6.3) in R (version 3.5.1) [49, 50], including correction for overdispersion. *P* value calculation was performed using the Chi-square test and *p* value correction for multiple testing was performed within each comparison using false discovery rate (FDR) [51, 52].

Spearman rank correlation analysis was performed to explore correlations between DNA methylation (LTR and *env*) and patient characteristics (HIV-1 reservoir and immunological parameters, obtained from Malatinkova et al*.* [39]). Therefore, methylation data of both regions of every individual was summarized by calculating an *M* value over all CpGs using the formula as described by Du et al. [53]. Using stepwise regression model selection, linear regression models were developed for LTR and *env* methylation densities to determine which independent variables may explain variable DNA methylation in both regions.

Visualization was performed using R (version 3.5.1) with the following packages: PMCMR (version 4.3), Hmisc (version 4.2-0), graphics (version 3.5.1), ggplot2 (version 3.1.0), and corrplot (version 0.84) [50].

## Results

### In silico, in vitro, and in vivo HIV-1 DNA methylation assay development

**Fig. 2:**
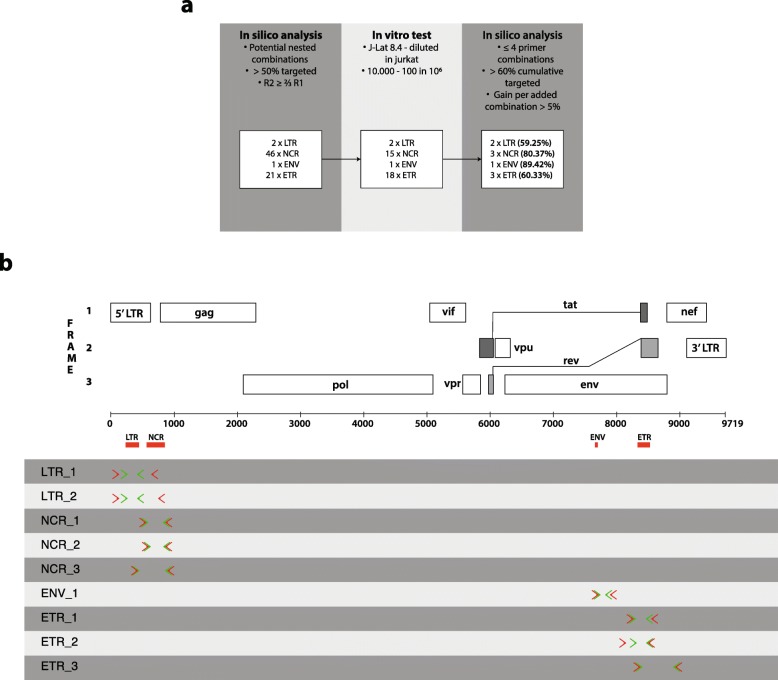
Primer selection procedure. **a** Workflow used for the development of our DNA methylation assay determining HIV-1 DNA methylation in HIV-1-infected patient samples. **b** Location of the nine different assays on the HIV-1 genome. Red arrows depict first round PCR primer location, green arrows show second round PCR primer location, red bars indicate the location of the four analyzed CpGIs based on Chavez et al. [12]

Three hundred thirty-eight different nested primer combinations (assays) (13 LTR, 303 NCR, 1 ENV, and 21 ETR) were subjected to an in silico analysis using an adapted version of the bioinformatics tool developed by Rutsaert et al. [46] to estimate the complementarity to the Los Alamos National Library database, resulting in 70 nested PCR assays (2 LTR, 46 NCR, 1 ENV, and 21 ETR, Fig. 2a). The performance of these assays was subsequently tested by PCR amplification in undiluted and diluted J-Lat 8.4 DNA (up to 100 infected cells/10^6^ cells), resulting in 36 assays (2 LTR, 15 NCR, 1 ENV, and 18 ETR) that were capable of generating PCR products at the lowest dilutions (Fig. 2a). After a final in silico analysis, a set of 9 primer combinations (2 LTR, 3 NCR, 1 ENV, and 3 ETR; Fig. 2 and Additional File 1) was selected.

These nine assays were used to determine the HIV-1 methylation profile of HIV-1-positive blood samples. The percentage of patients for whom the primer combinations generated PCR amplicons is listed in Table 2. This data demonstrates a similar trend as expected based on the in silico analysis, being that a certain percentage of HIV-1 sequences would not be detected in patients for certain primer combinations due to HIV-1 sequence variation. The difference between expected amplification percentage and the actual amplification percentage was 7.85%, 1.57%, 10.58%, and 3.57% for LTR, NCR, ENV, and ETR, respectively (Table 2).

**Table 2:** Performance of the nine final assays compared with the predicted performance using in silico analysis of the primer complementarity

	LTR	NCR	ENV	ETR
# primer combinations	2	3	1	3
% of patients expected to generate amplicons based on in silico analysis^*^	59.25	80.37	89.42	60.33
% of patients in which DNA was amplified	51.40	81.94	100	63.90
% of patients for which the DNA had sufficient quality to be mapped to HIV-1	48.61	75.00	41.67	63.90

^*^In silico analysis is based on the bioinformatics primer evaluation tool as described by Rutsaert et al. [46]

### SRCV shows increased LTR methylation and decreased *env* methylation

**Fig. 3:**
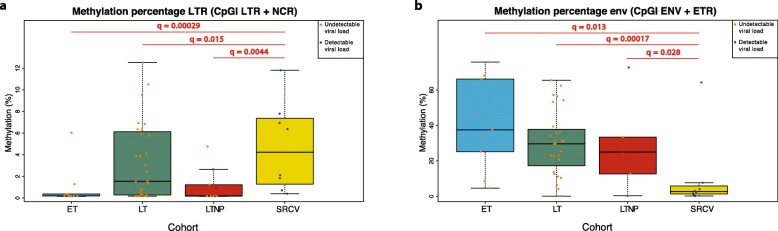
HIV-1 proviral DNA methylation comparison between patient cohorts. **a** Summary of the methylation data in the LTR region (CpGI LTR + CpGI NCR) using average methylation over all CpGs in the region. **b** Summary of the methylation data in the *env* region (CpGI ENV + CpGI ETR) using average methylation over all CpGs in the region. *q* = FDR-corrected *p* values for multiple testing. LT late treated, ET early treated, SRCV acute seroconverter, LTNP long-term non-progressor

In all four patient cohorts together, average methylation of all CpGs within the LTR region was 2.94% (IQR, 0.19–5.5%). When comparing patient cohorts, we observed significantly higher LTR methylation in SRCV as compared with all the other cohorts (ET, LT, and LTNP) (Δ = 6.48%; *q* = 0.00029, Δ = 4.15%; *q* = 0.015, and Δ = 5.94%; *q* = 0.0044, respectively) (Fig. 3a).

Higher CpG methylation was observed in the *env* region as compared with LTR, averaging 28.86% (IQR, 8.73–39.44%). All cohorts (ET, LT, and LTNP) showed a significantly higher methylation density compared with SRCV (Δ = 33.47%; *q* = 0.013, Δ = 35.32%; *q* = 0.00017, and Δ = 35.26%; *q* = 0.028, respectively) (Fig. 3b).

### Correlations between HIV-1 methylation status and reservoir markers

**Fig. 4:**
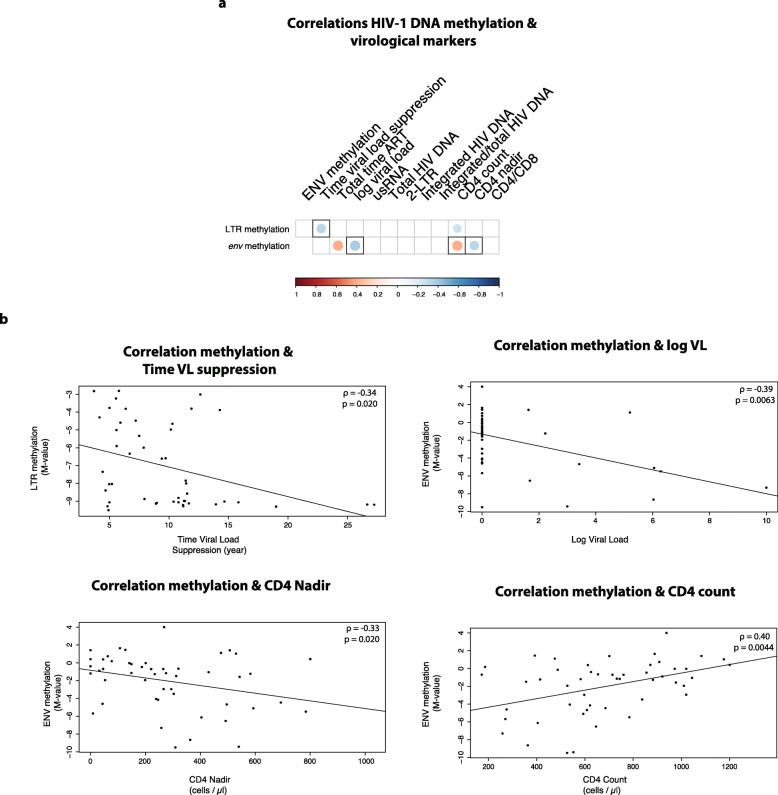
Spearman correlations between HIV-1 proviral DNA methylation and patient characteristics. **a** Correlation of DNA methylation with several virological and viral reservoir markers in HIV-1-infected individuals. Positive and negative correlations are depicted in red and blue, respectively. Non-significant correlations are left blank. Correlations with covariates that independently explained methylation in the linear regression models are depicted with a black frame. **b** Correlation plots between DNA methylation (*M* value) and the independent variables from the linear models. Upper left, LTR methylation vs. duration of VL suppression. Upper right, *env* methylation vs. log VL. Lower left, *env* methylation vs. CD4 nadir. Lower right, *env* methylation vs. CD4 count

During the explorative correlation analysis, negative correlations were found between the DNA methylation density in the LTR region and the duration of viral suppression (*ρ* = − 0.34; *p* = 0.020) and CD4+ T cell count at time of collection (*ρ* = − 0.27; *p* = 0.043) (Fig. 4a). However, we observed a significantly positive association for DNA methylation in the *env* region and the CD4 T cell count (*ρ* = 0.40; *p* = 0.0045) and cART duration (*ρ* = 0.39; *p* = 0.0055) (Fig. 4a). Moreover, *env* methylation decreased with increasing VL levels (*ρ* = − 0.39; *p* = 0.0063) and higher CD4+ T cell nadir (*ρ* = − 0.33; *p* = 0.020) (Fig. 4a). Based on the linear regression models, the only variable that was independently associated with DNA methylation in the LTR was the duration of VL suppression. Three variables were independently associated with the *env* methylation: VL, CD4 nadir, and CD4 count at time of sampling (Fig. 4b).

## Discussion

The lack of consensus about the role of proviral DNA methylation in HIV-1 transcriptional regulation illustrates the need for a reliable and widely applicable methylation assessment method. In this study, we first described an in silico procedure to accurately predict the complementarity of PCR assays to the HIV LANL database, and an in vitro validation protocol to test the sensitivity of the designed assays. This procedure resulted in nine functional DNA methylation assays, designed against the four most common CpGIs of the HIV-1 provirus, which were consequently used to characterize HIV-1 DNA methylation in a large, well-characterized patient cohort. The in silico analysis was predictive of the number of patient samples leading to successfully amplified PCR products (Table 2), indicating that this is an effective approach to prioritize testing of primer sets in the context of HIV-1 or other pathogens with a high sequence variability. In addition, as shown in the study of Cortés-Rubio et al. [14], by using an NGS-based approach, our method fulfills the need to analyze a large number of proviruses for each patient when compared with the established Sanger sequencing-based methods [54].

Across our four patient cohorts, we have found that the HIV-1 provirus had low amounts of DNA methylation in the promoter region (average 2.94%, IQR 0.19–5.5%) but substantially higher levels of intragenic (*env*) methylation (average 28.86%, IQR 8.73–39.44%). When comparing the differential methylation between the cohorts, only SRCV showed distinct methylation profiles, with increased LTR, and decreased *env* methylation. Similarly, if patients were divided based on their VL status (detectable VL (VL > 40 HIV-1 copies/ml plasma), comprising all SRCV and 6/17 LTNPs. vs. undetectable VL (VL < 40 HIV-1 copies/ml plasma), comprising ET, LT, and 11/17 LTNPs), individuals with a detectable VL had higher DNA methylation density in the HIV-1 LTR region and a lower density in the *env* region compared with those with an undetectable VL. These observations might indicate that specific methylation profiles may be associated with in vivo HIV-1 transcriptional control and latency maintenance.

Indeed, since the involvement of DNA methylation in HIV-1 latency was first described in 1987 [55], it has been confirmed in HIV-1-infected cultured cells and latency models that promoter methylation density is associated with silencing stability: DNA methylation induction can initiate/stabilize HIV-1 latency, while methylation inhibitors as 5-aza-2′-deoxycytidine (5-aza-CdR) cause HIV-1 reactivation and display clear synergistic effects with other latency reversing agents [11–13, 32–34, 36, 56–58]. These studies reported a major role of promoter DNA methylation in latency regulation, which was in line with the general concept of transcription regulation by DNA methylation: hypermethylation of the promoter region suppresses both basal promoter activity and responses to activating stimuli, and hypomethylation is a transcription mark [57]. However, DNA methylation studies on patient-derived samples have shown—with the exception of some LTNPs—the same trend as in our present observation: low level of DNA methylation in the promoter region, even in patients suppressing VL successfully, therefore not following the predictions from the in vitro experiments [37, 38]. It has been shown that DNA methylation behavior in cell lines is often drastically different from that of in vivo cells due to completely different epigenetic environments and immortalization, sometimes producing unreliable results in terms of predicting in vivo DNA methylation events [59, 60]. Some studies, however do show increasing LTR DNA methylation over time [13], or dynamic profiles in patients when measured longitudinally [14]. We could not confirm these data since we only measured single time point samples of patients with similar treatment time/time of virological control (except for the SRCV). The low abundance of DNA methylation in the promoter region of HIV-1 indicates that other (epigenetic) factors as integration site epigenetics or cell type might be more important for transcriptional regulation than promoter methylation.

In previous DNA methylation studies in HIV-1 patients, the focus was on promoter methylation assessment [13, 14, 32, 36–38]. In contrast to promoter methylation, the role of intragenic DNA methylation in general transcriptional regulation is less clearly described [26–30]. Studies outside of the HIV-1 field have suggested that intragenic methylation could have a role in the activation of retroviruses, repetitive elements, alternative splicing, transcription initiation in canonical promoters of embryonic stem cells, and prevention of aberrant transcript production [28–30]. Moreover, intragenic methylation has been shown to be a robust predictor of gene transcription in genes with a CpGI containing promoter [61]. In our study, decreased *env* methylation levels in individuals with active ongoing replication (SRCVs) suggests that intragenic methylation increases in the case of proviral transcriptional silencing, leading to higher methylation in latently infected cells or in those in which viral replication is blocked. Indeed, cART-treated patients and LTNP have lower viral transcription (measured as cell-associated unspliced RNA (CA usRNA)) than SRCV (Table 1) and *env* methylation shows an inverse correlation with CA usRNA within the SRCV cohort (*ρ* = − 0.81; *p* = 0.014). Furthermore, intragenic methylation did correlate positively with the CD4+ T cell count, linking high intragenic methylation with viral control. Intragenic methylation was also negatively associated with the VL, a measure that indicates ongoing replication.

In contrast to what was proposed by LaMere et al*.* [54], we have found no statistical difference between proviral methylation in LTNP with undetectable VL (latent infection) and treated patients (cART-induced suppression) (LTR: Δ = 0.85%, *q* = 0.74; *env*: Δ = 2.29%, *q* = 0.94). This could be due to the low number of LTNPs with undetectable VL.

In general, the lack of promoter DNA methylation in HIV-1 proviral genomes in vivo suggests that this modification is of subordinate importance in the regulation of the viral life cycle compared with the more abundant, yet less studied intragenic DNA methylation. Our observations indicate that intragenic DNA methylation could be a late event during infection. Methylation of the proviral genome may occur stochastically during years of viral control, yet act as a stable epigenetic mark once established. This may subsequently affect transcription, including splicing, of viral transcripts, which could affect viral replication by interaction with transcriptional elongation (tat) or export of viral RNA (rev). Nevertheless, additional in vitro and in vivo experiments targeting the (intragenic) DNA methylation are required to evaluate the exact impact on the HIV-1 life cycle. Especially temporal changes of intragenic methylation would be very informative, yet our study was limited by the lack of longitudinal sampling. Other limitations include the fact that although the cohort size was much larger than previous studies [13, 14, 32, 36–38], the patient groups described here were not balanced, not in size, nor for sex, and age. Additionally, we did no specific CD4+ T cells selection. The use of PBMCs could potentially mask differential methylation since it is shown that LRAs have cell-type specific effects, indicating cell-type specific epigenetic profiles [62]. Moreover, due to the targeted nature of the methodology, it does not allow to provide information about integration site methylation or replication competence of the analyzed provirus. Finally, we did not provide information about the fifth CpGI (3′ LTR), nor did we analyze non-CpGI CpGs.

## Conclusions

Altogether, our study illustrates the underestimation of the role of intragenic proviral DNA methylation in patient samples. Previous studies have mainly focused on LTR methylation and have interpreted LTR methylation as a transcriptional regulatory factor, ignoring any potential role of *env* methylation [13, 35, 38]. We suggest that both *env* and LTR methylation are involved in HIV-1 transcription regulation and that *env* methylation could be an important predictor of viral transcription in vivo. However, we also suggest that proviral promoter methylation is hindered/inhibited in all HIV-1-positive patients, especially those on cART, but that its density still influences viral transcription rate.

The exact functions of DNA methylation of these two regions should be clarified by performing additional experiments using longitudinal follow-up studies to monitor proviral DNA methylation dynamics within patients, starting early during infection, and ideally continuing over a period of multiple years of cART. Different CD4+ T cell types should be analyzed separately to avoid cell-type dependent bias of the data. If HIV-1-positive patients were to undergo treatment interruption, DNA methylation profiles should also be monitored in order to understand the methylation dynamics during viral rebound. Moreover, proviral intragenic non-CpGI methylation analysis could also provide a better understanding of HIV-1 latency regulation by DNA methylation. Here, we do provide a useful tool to help design and estimate the sample size needed in these studies. Altogether, these insights should be of paramount importance when looking at the various strategies to control HIV-1 after discontinuation of cART and for the HIV-1 cure field.

## Supplementary information

**Additional file 1.** Primers and PCR experiments.

**Additional file 2:Figure 1.** Overview of patient cohorts included in this study. Patients are divided into four groups based on their disease state: early treated, late treated, Long-Term Non-Progressor and acute seroconverter. Arrows depict moment of sampling. PHI = Primary HIV-1 Infection; cART = combination Anti-Retroviral Therapy.

## Data Availability

Datasets used during the current study are available from the corresponding author on request.

## Abbreviations

5-aza-CdR: 5-aza-2′-deoxycytidine
AIDS: Acquired immunodeficiency syndrome
CA usRNA: Cell-associated unspliced RNA
cART: Combination antiretroviral therapy
CpGIs: CpG islands
ET: Early cART-treated individuals
ETR: *env*-*tat*-*rev*
HIV-1: Human immunodeficiency virus type 1
IQR: Interquartile range
LANL: Los Alamos National Laboratory
LT: Late cART-treated individuals
LTNP: Long-term non-progressors
LTR: Long terminal repeat
NCR: Non-coding region
NGS: Next-generation sequencing
PBMC: Peripheral blood mononuclear cells
qPCR: Quantitative real-time PCR
VL: Viral load
