# Supplementary material for: Underestimated effect of intragenic HIV-1 DNA methylation on viral transcription in infected individuals
Source: Clin Epigenetics. 2020 Feb 28;12:36. doi: 10.1186/s13148-020-00829-1 (PMC7049218; doi:10.1186/s13148-020-00829-1)
Supplement: Supplementary file 1 — Additional file 1. Primers and PCR experiments. [file 13148_2020_829_MOESM1_ESM.pdf]

Supplementary file 1: Primers PCR experiments

	Sequence Round 1	Binding region (HXB2)	Name Sam	Protocol round 1
**LTR**				
**Round1**				
LTR_1_f (a)	TAGATATTTATTGATTTTTGGATGGTG	110-686	ART2-ART3	Trejbalova_R1 (a)
LTR_1_r (a)	AAAAAACTCCTCTAATTTYHCTTTC			
LTR_2_f (a)	TAGATATTTATTGATTTTTGGATGGTG	110-796	ART2-ART2	Trejbalova_R1 (a)
LTR_2_r (a)	CACCCATCTCTCTCCTTCTAACCTC			
**Round2**				
LTR_1_f (a)	AGTGTTAGTGTGGAGGTTTGATA	248-464	ART2-ART3 – ART3-EXTRA	Trejbalova_R2 (a)
LTR_1_r (a)	CAAAAAAACCCAATACAAACAAAAA			
LTR_2_f (a)	AGTGTTAGTGTGGAGGTTTGATA	248-464	ART2-ART2– ART3-EXTRA	Trejbalova_R2 (a)
LTR_2_r (a)	CAAAAAAACCCAATACAAACAAAAA			
% expected amplicon (*in silico* analysis)	59.25			
% amplified	51.4			
**NCR**				
**Round1**				
NCR_1_f	TGTGTGATTTTGGTAATTAGAGATTT	572-910	LTR02	Meth56
NCR_1_r	ACTCCCTACTTACCCATACTATAT			
NCR_2_f	TAGTGTGGAAAATTTTTAGTAGTGG	614-874	M3L7	Meth55
NCR_2_r	ATTATTTCTTTCCCCCTAACC			
NCR_3_f	TGTATATAAGTAGTTGTTTTTTGTTTGT	423-914	M2L4	Meth56
NCR_3_r	TCTAACTCCCTACTTACCCATACTATA			
**Round2**				
NCR_1_f	TGTGTGATTTTGGTAATTAGAGATTT	572-871	LTR02 -> L2L15	Meth55
NCR_1_r	ATTTCTTTCCCCCTAACCTTAAC			
NCR_2_f	TGTGGAAAATTTTTAGTAGTGG	617-874	M3L7 -> LTR07	Meth54
NCR_2_r	ATTATTTCTTTCCCCCTAACC			
NCR_3_f	TGTATATAAGTAGTTGTTTTTTGTTTGT	423-912	M2L4 -> Z2M2	Meth57
NCR_3_r	TAACTCCCTACTTACCCATACTATATA			
% expected amplicon (*in silico* analysis)	80.37			
% amplified	81.94			
**ETR**				
**Round1**				
ETR_1_f (b)	AGTGAATAGAGTTAGGTAGGGATATT	8336-8644	ETR02	Weber (b)
ETR_1_r (b)	AATTCCTAACTCCAATACTATAAAAA			
ETR_2_f	GTAAAATTAGTAAGAAAAGAATGAA	8171-8557	ETR11	Weber (b)
ETR_2_r	ACAATCAAAAATAAATCTCTCAAAC			
ETR_3_f	GTGGAGAGAGAGATAGAGATAGAT	8434-9000	ETR07	Trejbalova_R1 (a)
ETR_3_r	AAAACCCACCTCCTCCTC			
**Round2**				
ETR_1_f (b)	GAGTTAGGTAGGGATATTTATTATT	8344-8559	ETR02 -> ETR06	Weber (b)
ETR_1_r (b)	TTACAATCAAAAATAAATCTCTCAA			
ETR_2_f	TTGTATTTTTTATAGTGAATAGAGTTA	8323-8557	ETR11 -> ETR10	Weber (b)
ETR_2_r	ACAATCAAAAATAAATCTCTCAAAC			
ETR_3_f	GTGGAGAGAGAGATAGAGATAGAT	8434-8989	ETR07 -> ETR08	Trejbalova_R2 (a)
ETR_3_r	CCTCCTCCTCTTATACTTCTAACC			
% expected amplicon (*in silico* analysis)	60.32			
% amplified	63.9			
**ENV**				
**Round1**				
ENV_1_f	AGTTTTGTTTTTTGGGTTTTTG	7772-7980	ENV1	Trejbalova_R1 (a)
ENV_1_r	ATCTTTCCACAACCAAAATTCT			
**Round2**				
ENV_1_f	AGTTTTGTTTTTTGGGTTTTTG	7772-7905	ENV1 -> ENV2	Trejbalova_R2 (a)
ENV_1_r	CCTCAATAACCCTCAACAAATTA			
% expected amplicon (*in silico* analysis)	89.42			
% amplified	100			

Supplementary file 1: Primers PCR experiments, continued

Trejbalova_R1 (a)

	Temperature (ºC)	Time (min:sec)	Cycles
Initial denaturation	94	5:00	1
Denaturation	94	0:15	
Annealing	65 - 1 / 2 cycles	1:30	20
Elongation	72	1:00	
Denaturation	94	0:15	
Annealing	57	1:30	20
Elongation	72	1:00	
Final elongation	72	7:00	1

Trejbalova_R2 (a)

	Temperature (ºC)	Time (min:sec)	Cycles
Initial denaturation	94	5:00	1
Denaturation	94	0:15	
Annealing	58	1:30	20
Elongation	72	1:00	
Denaturation	94	0:15	
Annealing	65	1:30	20
Elongation	72	1:00	
Final elongation	72	7:00	1

Weber (b)

	Temperature (ºC)	Time (min:sec)	Cycles
Initial denaturation	95	5:00	1
Denaturation	95	1:00	
Annealing	58	2:00	40
Elongation	72	2:00	
Final elongation	72	2:00	1

Meth

	Temperature (ºC)	Time (min:sec)	Cycles
Initial denaturation	95	5:00	1
Denaturation	95	0:15	
Annealing	Temp + 4	1:30	5
Elongation	72	1:00	
Denaturation	95	0:15	
Annealing	Temp + 2	1:30	5
Elongation	72	1:00	
Denaturation	95	0:15	
Annealing	Temp	1:30	40
Elongation	72	1:30	
Final elongation	72	7:00	1

(a) Trejbalová et al., 2017 [13]
(b) Weber et al., 2014 [37]
